# “It’s adjusting the mindset”: physical activity participation during chemotherapy

**DOI:** 10.1007/s00520-026-10628-8

**Published:** 2026-04-09

**Authors:** Bertram J. J., Pickens N., Goh H. T.

**Affiliations:** 1 School of Physical Therapy, Texas Woman’s University, Dallas, TX USA; 2 Outpatient Division, Baylor Scott and White Institute for Rehabilitation, Grapevine, TX USA; 3 Texas Woman’s University, Denton, TX USA

**Keywords:** Breast neoplasm, Exercise, Patient experience, Quality of life, Qualitative research

## Abstract

**Purpose:**

Chemotherapy is associated with multiple side effects (e.g., fatigue, functional decline, emotional distress), but research suggests that the side effects can be mitigated or prevented through regular engagement in physical activity. Yet, there is a known decline in physical activity during chemotherapy. Understanding what a woman affected by breast cancer experiences during chemotherapy may provide insight into ways to increase physical activity engagement. This study explores the experience of women with breast cancer receiving chemotherapy and how they navigate physical activity participation during chemotherapy.

**Methods:**

Ten semi-structured qualitative interviews were conducted with women receiving chemotherapy for the treatment of breast cancer from June to October 2024.

**Results:**

The results were synthesized using a reflexive thematic analysis approach; identifying one overarching theme and four sub-themes. The four sub-themes provided specific insights into why physical activity participation was experienced differently during chemotherapy. Going through chemotherapy changed how the women spent their time, how they interacted with people around them, and the roles they fulfilled. Chemotherapy exposed simple tasks (e.g., shower, walking upstairs) that the women previously did not consider physical activity, as activities that expended a significant amount of energy. During chemotherapy, each day felt different so the decision to participate in physical activity balanced on how the women felt, or anticipated feeling, and the perceived benefits or risks of participating.

**Conclusion:**

Healthcare providers have new insights from women with breast cancer to best recommend relevant and achievable physical activity goals and help address physical activity deficits during chemotherapy.

**Supplementary Information:**

The online version contains supplementary material available at 10.1007/s00520-026-10628-8.

## Introduction

The American Cancer Society has established that regular physical activity, > 150 minutes of moderate- or 75 minutes of vigorous-intensity physical activity per week, is safe and beneficial for persons undergoing breast cancer treatment [1]. Research shows that regular physical activity makes a positive impact on patient functioning, quality of life, treatment tolerance, and life expectancy [2–7]. Despite strong evidence supporting the clinical benefits, participation in physical activity following a cancer diagnosis is low [4, 5, 8–11]. Participation in physical activity further declines during chemotherapy but little is known how chemotherapy influences physical activity participation as the experience during chemotherapy is highly individualized [5, 9, 12]. Exploring the individual experience using a qualitative approach may provide a richer understanding of how treatment-related symptoms affect individuals and their physical activity participation.

Previous qualitative studies found that symptom burden [13–17], lack of motivation [18, 19], lack of knowledge about physical activity [13, 16, 20–22], and time constraints [18, 20] are barriers to participation in physical activity during and after cancer treatment. In contrast, facilitators to participation included an enhanced sense of well-being [14, 16, 18, 20], a belief that exercise made them feel better [23, 24], and a feeling of purpose [17, 20]. However, these qualitative studies were done within the context of an intervention study where a supervised exercise program was prescribed [17, 18, 23], included a mix of cancer types [15, 16, 24], excluded breast cancer [14], and included a mix of treatment phases (during and after chemotherapy) [20, 22]. Women who undergo chemotherapy for breast cancer present with unique side effects (e.g., hormone deprivation, bone demineralization, myalgias/arthralgias, increase in fat-to-muscle ratio) that are not present in other leading types of cancer, such as lung and colon. The majority of these side effects can be mitigated or improved by regular physical activity, but the available literature does not provide a picture of what is experienced by community-dwelling women who undergo chemotherapy and how their experience affects physical activity participation. The individual experience of women during chemotherapy for breast cancer is largely under-explored and may provide insight into the physical factors (e.g., fatigue, pain, body weight), emotional factors (e.g., fear, motivation, depression), and environmental factors (e.g., time, support) that influence the decision to engage in physical activity [15, 18, 20, 25].

The purpose of this study was to explore, through semi-structured interviews, what women experienced during chemotherapy, specifically what they thought and felt about physical activity during chemotherapy. The research question for this study was “What is the experience of women with breast cancer in navigating physical activity participation during chemotherapy?”

## Methods

### Design

A qualitative approach with semi-structured interviews and Braun and Clark’s method of thematic analysis [26] was best suited to gain a comprehensive understanding of the complex feelings, impressions, and beliefs held by women receiving chemotherapy, which is vital to engage community-dwelling women towards physical activity engagement.

### Recruitment

Participants included a convenience sample of women from prior research [27]. Eligibility criteria included females recently diagnosed (≤ 6 months) with stage I–III breast cancer who were actively receiving chemotherapy at the time of data collection, fluent in the English language, over 18 years old, and capable of participating in physical activity as determined by a screening questionnaire. Written and verbal consent was obtained before the interview started. Pseudonym initials are used throughout.

### Data collection

Semi-structured interviews were conducted on Zoom (Zoom Video Communications, Inc.) using an interview guide (see Supplementary Information). The interview guide included a reflexivity activity, interview questions, and post-interview reflection prompts. During the interviews, the open-ended questions were drawn from the interview guide but remained flexible and adaptable based on the participant response to encourage participants to elaborate on their feelings, thoughts, and experiences and to ensure depth of understanding as the interviewer. Interviews were audio-recorded and ranged from 50 to 60 minutes.

### Data analysis

Interview transcription and data analysis were done concurrently with data collection. Interviews were transcribed initially by Zoom and then de-identified and checked for accuracy by one author (J.J.B) before digitally stored on a secured drive and uploaded to NVivo (Lumivero) for analysis. The study applied a thematic analysis approach to identify patterns (or themes) that illuminated the thoughts, feelings, and actions of women and their decision to participate in physical activity [26, 28]. Table 1 summarizes the steps involved and contributing authors in the data analysis.

**Table 1:** Summary of Braun and Clarke’s six-step process for thematic inductive analysis [26]

Initial reading of the transcripts	Transcriptions were read and re-read in detail and writing was employed as an analytic tool to develop coding ideas (J.J.B)
Initial coding of the data	Similarities and differences were identified among participants’ responses to establish an initial coding frame (J.J.B)
Themes and subtheme development	The common codes and supporting data were organized into themes and subthemes (J.J.B and N.P)
Review theme and subthemes	The themes and subthemes were reviewed with consideration to the initial codes and entire data set. A thematic map of the analysis helped gain a deeper insight into the developing themes and subthemes (all authors)
Fortification of the themes and subthemes	The interviews were re-read to ensure no new themes or supporting data were identified (J.J.B). A tenth interview was conducted after data saturation to ensure saturation (J.J.B). Before the themes were finalized, all participants were invited to participate in a second interview to confirm the authenticity of the results. Four participants completed the member checking interview. During the second interview, participants were asked to critique the thematic structure and content, and provide any feedback, suggestions, or alternative perspectives on the data (J.J.B). The women who participated in the second interview were unanimous in their support of the established thematic map, supporting extracts, and the graphic illustration
Selection of supporting examples	The names for the overarching theme and themes were finalized and specific quotes were identified that accurately depicted the data and common themes (all authors)

To ensure trustworthiness of the findings, an audit trail was organized using the six phases of data analysis and included a checklist for accountability and enhanced rigor [26, 28, 29]. Reflexive journaling was employed throughout to minimize bias and enhance sincerity of the findings [30]. Reflexive prompts were addressed by the first author prior to each interview including bias towards physical activity during chemotherapy and how that influences beliefs about patient experiences. These reflections, and acknowledging experts also have bias, were critical to keeping an open mind during the analysis towards the individual and collective experiences of these women. Deep knowledge and understanding support a rich understanding while holding bias in check. Member-checking was conducted with four participants on Zoom during which the participants were provided a summary of the research findings and were asked to comment on or question any aspect of the research and assure the participants’ experiences were accurately represented [28–30]. The initial, developing and final themes were reviewed by a qualitative expert (N.P.) to add validity and credibility to the findings [26, 28].

## Results

Ten interviews were conducted between June 2024 and October 2024. Participant demographics are summarized in Table 2.

**Table 2:** Participant demographics

	*N* (%) participants, *n* = 11
**Age, years**	
30–39	3 (30)
40–49	4 (40)
50–59	2 (20)
60–69	1 (10)
**Marital status**	
Never married	2 (20)
Married	8 (80)
**Education**	
2-year college	1 (10)
4-year college	4 (40)
Professional degree	4 (40)
Doctorate	1 (10)
**Region**	
Northeast	2 (20)
Midwest	2 (20)
South	4 (40)
West	2 (20)
**Race**	
White	6 (60)
Black or African American	2 (20)
Hispanic	2 (20)
**Community setting**	
Urban	2 (20)
Suburban	8 (80)
**Cancer stage**	
Stage I	3 (30)
Stage II	5 (50)
Stage III	2 (20)

### Overarching theme: “it’s adjusting the mindset”

The overarching theme, “it’s adjusting the mindset,” is central to how the women navigated physical activity during chemotherapy. The changes in their mindset revolved around what physical activity looked and felt like during chemotherapy, how their body felt and responded to physical activity, and how they prioritized activities during chemotherapy. As LM shared, “There’s just something when you go to the gym and you work out. You just, even though I can’t do what I used to do, I just feel better. It psychologically makes you feel better and your body does feel better. You may, you may tire easy, but overall, then your body feels better. So it’s, it’s adjusting the mindset.” The adjustment of mindset was two-fold: acceptance of current level of physical ability and adjusting the type of activity and intensity. The change in mindset was a conscious decision, belief, or attitude that recognized physical activity was important even when their body felt and responded differently. The following four subthemes, “definitely a change in perspective,” “the price to pay,” “trying to retain a sense of who I am,” and “making a conscious choice about each thing,” provide an in-depth explanation of the experience in navigating physical activity participation during chemotherapy. Each subtheme highlights a particular aspect of how participants reoriented their thinking and actions while navigating physical activity participation during chemotherapy.

#### Theme 1: “definitely a change in perspective”

The theme, “definitely a change in perspective,” encompasses the mental and physical changes that occurred during chemotherapy and how those shifts influenced physical activity participation. Several women shared that going through chemotherapy forced them to realign their daily priorities. AC shared, “So in the beginning I would try to prioritize other things like, ‘Okay, let me, maybe, let me do the dishes.’ … And what I came to realize was I needed the most attention and I needed to sit down somewhere so everything gets tabled now…. Now it’s like, no, I prioritize myself first.” The side effects of chemotherapy necessitated rest and time for personal renewal and rejuvenation, which in turn limited their capacity for daily activities and resulted in a flip of their priorities and their roles within their home and community.

Participants also changed their perspective on what activities constituted physical activity. All the women were asked to define physical activity in their own words and there was a general consensus that physical activity was exercise, such as hiking, biking, or swimming. However, during chemotherapy, physical activities were experienced differently. For example, SB shared that prior to chemotherapy, many of the activities she performed did not feel like physical activity, “[T]here were no limitations on walking and things like that if I wanted to go to the fair or take the dog for a walk or something like that. I guess I didn’t think about that as activity previously because those types of activities were very normal.” However, during chemotherapy, many simple tasks were daunting and, in some cases (e.g., going to the fair), they were unable to participate in at all. While the definition of physical activity did not change for the women, the way they experienced activity did.

#### Theme 2: “the price to pay”

The theme, “the price to pay,” captured how the women described the side effects of chemotherapy and the physical activities that were affected by chemotherapy. Chemotherapy affected women physically (fatigue, pain, nausea) and mentally (worry, chemo brain, anxiety), limiting their ability to participate in a range of physical activities. The severity of life disruption varied from person to person, but there was a common sentiment that the physical and mental side effects were worth suffering if the chemotherapy killed the cancer. MW shared, “I just wanna get through, and just have, you know, a good outcome and get past this, that's all. It's been a journey, and I don’t regret going through it. I just want to get through it.” For MW, and others, the journey through chemotherapy was purposeful and approached with a goal in mind. The way they felt, the things they experienced, and the hardships they endured were accepted as part of the cancer treatment.

During chemotherapy, physical activity was perceived as important but came at a cost. SJ described the physical experience of participating in activity and how it made her feel, “It’s just, you’re just exhausted… And it’s mentally I want to, and it’s mentally I think it’s good to go out and be outside but it’s physically every muscle aches and my chest hurts.” The side effects from chemotherapy stripped SJ’s physical ability even when the mental determination remained. Similarly, other women shared that chemotherapy had cost them their ability to perform daily tasks such as showering, toileting, and preparing a meal. SB shares, “you know, just to walk to the bathroom – it’s something I will delay until I can’t delay it anymore because I know it’s gonna hurt that much. And that, that’s bonkers to think about.” Chemotherapy exposed simple tasks, that the women previously did not consider physical activity, as activities that were exhausting or draining.

#### Theme 3: “trying to retain a sense of who I am”

The third theme, “trying to retain a sense of who I am,” described the inherent desire for normalcy during a season of life that was unexpected and unpredictable. Going through chemotherapy changed how the women spent their time and the roles they fulfilled. The disruption in their normal roles and activities prompted many women to seek opportunities that felt normal. RH talked about how the activities around her job made her feel, “For me, [my job] has been a good escape, you know, it does give me something else to focus on besides, you know treatments or just being cancer person…It definitely gives me that getaway. And I’m really glad that I just have something where, again, cancer doesn’t quite touch it.” The idea of doing normal activities to feel like a normal person was present throughout all interviews. Whether it was working, going to social gatherings, or attending events with children, the women sought opportunities to engage in regular, daily activities.

Though maintaining normal activities and roles came at a cost (e.g., fatigue, pain), the negative experience did not override the benefits of feeling normal. One woman (SJ) shared her cost-benefit perspective, “I feel like even now I still try to live as normal a life as possible, even if I can only go somewhere for an hour or so, and I don’t think I would change that, because I think mentally it’s better for me. Even if it means I’m exhausted for the rest of the today I’d rather be exhausted having done something for an hour than tired but having done nothing.” This benefit over cost perspective extended to their roles in the home and community. MW discussed how making breakfast for her family was physically draining but the task made her happy and it made her family happy. She felt she was doing something she should be doing – fulfilling her role as a wife and mother by providing meals. Maintaining normal activities was important for all the women but the physical and mental demands of tasks required the women to choose what activities were prioritized.

#### Theme 4: “making a conscious choice about each thing”

The fourth theme, “making a conscious choice about each thing,” focuses on the decisions the women made around physical activity. There was a consensus that physical activity offered physical benefits (e.g., less fatigue, weight management, improved sleep) and positive psychological changes (e.g., decreased anxiety, enhanced mental well-being), but often, they experienced an internal struggle with participation. Several women felt that by choosing to participate in exercise they were compromising their ability to participate in other activities, such as social or family responsibilities. They opted to forgo certain physical activities to prioritize others. SB shared, “[B]efore, I was walking after dinner at night and doing, like, some gardening and some yard work…[Now,] I find myself, like, I don’t know, trying to conserve energy for when school gets out and I need to be there with the kids to do all the different things.” For SB and others, participation in physical activity was weighed against how they felt mentally and physically and what tasks they needed to prioritize.

The women shared that they only had a limited amount of energy available. Depending on the day, the women had to decide what takes priority and how much energy they were willing to spend. At times, the expenditure of energy on a certain task was at the expense of another. AC provided a creative illustration on energy expense, “I have like an energy bucket – so I have so much energy throughout the day. So it’s like, do I want to go and have a really great gym workout and then I leave all these other little tasks at home tabled or do I do all these little tasks, and now I have no energy to go to the gym.” Prior to chemotherapy, the women did not need to ration their energy or make conscious decisions about how they would spend time or energy. During chemotherapy, they considered the activity and the pros and cons of how they would feel following the activity. When making a conscious choice about each thing, the women were balancing their knowledge of the benefits of physical activity, their internal and external motivations for participating in physical activity, and their physical state.

## Discussion

 The majority of qualitative research on physical activity participation during breast cancer has been conducted in the context of an exercise intervention study or with participants attending a supervised exercise program [16–18, 23]. The present study included community-dwelling participants receiving chemotherapy for breast cancer, regardless of their prior or current level of physical activity. The women in our study defined physical activity similar to the operational definitions found in the research (any movement or activity that increases energy expenditure) [16, 22], but the activities that required significant energy expenditure changed with the introduction of chemotherapy. Prior to chemotherapy, the women described the activities that increased energy expenditure as physical exercise activities (e.g., hiking, running, swimming, cycling). Yet, during chemotherapy, the activities that increased their energy expenditure were not exercise but rather daily tasks (e.g., walking up stairs, walking the dog, taking a shower, cooking a meal). In this study, the definition of physical activity remained constant but the tasks that satisfied the definition changed. Recognition of this change in perception is paramount to understanding the decisions the women made around physical activity participation.

**Fig. 1:**
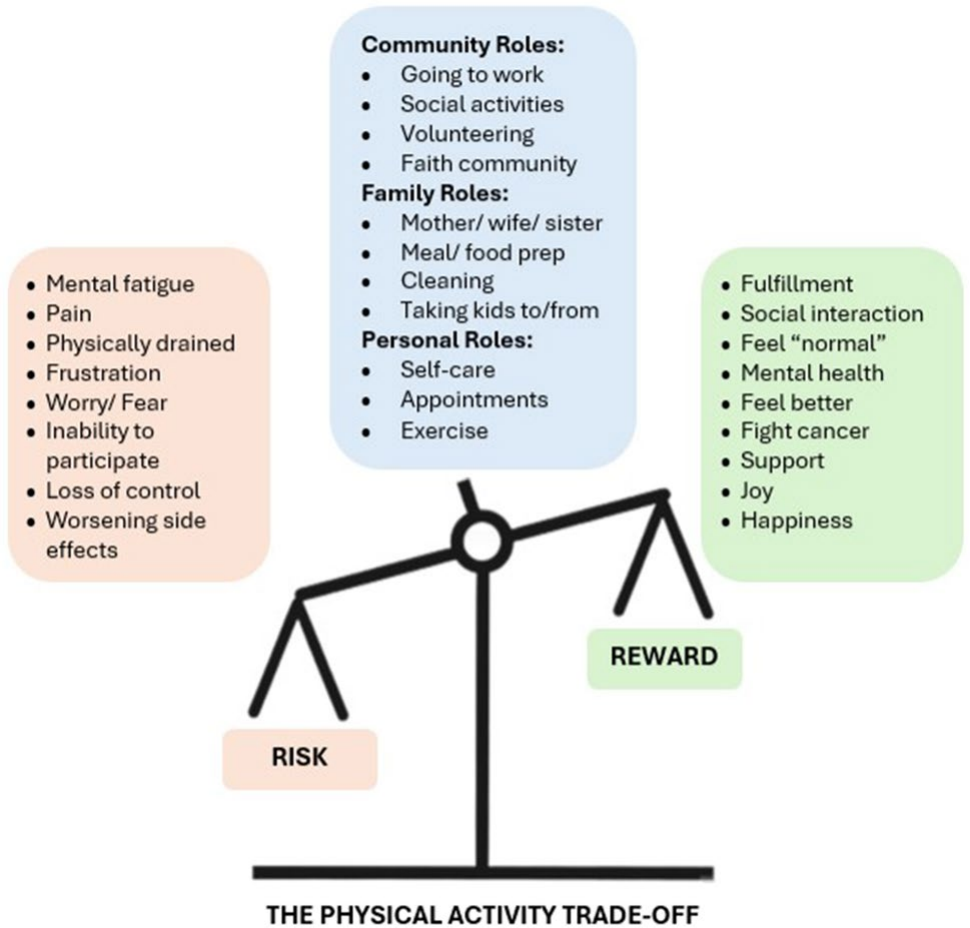
Visual interpretation of the physical activity trade-off for women receiving chemotherapy for the treatment of breast cancer

Our study revealed a complex, dynamic interaction among knowledge, motivation, and physical capability that drove the women’s decisions to participate in physical activity, echoing prior studies [18, 19, 31]. The graphic in Fig. 1 is a visual interpretation of choices made each day around physical activity. In the center of the graphic are the roles the women fulfilled and the activities they described in the interviews to retain a sense of normalcy (“trying to retain a sense of who I am”). The roles and activities hang in balance over a scale that measures risk (on the left) versus reward (on the right) for which they had to make conscious decisions to balance the scale (“making a conscious choice about each thing”). A shared observation among the women interviewed was that during chemotherapy, each day and each week look different—which is why the roles and activities are balancing on a scale that is volatile to change as their perspective for risk and reward changes constantly (“definitely a change in perspective”). There were days when running errands brought the woman joy because she felt normal and the task provided social interaction and fulfillment. On those days, the rewards for participating in physical activity were high and the risks low. However, on other days, running errands was mentally fatiguing, physically draining, and increased frustration or pain (“the price to pay”). This internal struggle between knowledge of the benefits, mental motivation, and physical restrictions varied day-to-day for the women. The variability in how the women felt mentally and physically during chemotherapy and the impact that had on physical activity is not well described in any previous literature. The results from this study explored that complex interaction and the factors that influenced decision making around physical activity participation.

The physical side effects related to chemotherapy treatment are documented as a barrier to physical activity participation [15, 16, 18, 32]. The women in this study confirmed that experiencing side effects (pain, fatigue, decreased stamina) made participation in physical activities more difficult. However, they felt the side effects were worth enduring because the chemotherapy was working to kill the cancer. This acceptance, and even welcoming, of side effects is not documented in the literature, to our knowledge, and provides a unique insight into a commonly cited barrier to physical activity participation. For many women, the exertion required to participate in daily activities (e.g., cooking, running errands, walking in the park with friends) increased pain and fatigue, but they viewed the activity as important and accepted the side effects. For others, the side effects were seen as a sign that the chemotherapy drugs were working and the side effects served as a motivation to push forward and through the discomfort. These results demonstrate the complexity of physical activity participation during chemotherapy and may shed light on the inconsistency in the literature. Individual perception of the side effects may have a strong influence on participation decisions.

The study findings indicated the decision to participate in physical activity was often influenced by social roles and the sense of normality that physical activity provided, which was consistent with previous research that described physical activity as a conduit for returning back to normal [16–20, 23]. The connection between physical activity and normality was present in this study but experienced at a deeper level than described in previous research. The women in this study shared that chemotherapy had threatened their identity, their priorities, and their values. The decisions around physical activity participation were driven from a place of feeling whole when cancer and cancer treatment threatened who they were and where they belonged. The physical activity tasks the women highlighted around maintaining normality were deeply connected to their identity and social support. Because the women often chose to prioritize physical activity tasks related to work, friends, or family, they were unable to participate in other activities, such as exercise. This prioritization of tasks during chemotherapy may explain the overall decline in physical exercise participation that is documented in the research [5, 9, 10]. The findings support a more holistic approach to physical activity education and guidance by healthcare professionals that is geared towards an individual’s unique roles and responsibilities in the home and community.

## Strengths and limitations

The women were recruited from the community and were unaffiliated with any group, organization, or facility, so the results represented a real-world perspective on navigating physical activity participation during chemotherapy. Additionally, the women had diverse demographics and varied levels of reported previous and current physical activity levels, which provided a more comprehensive understanding and helped reduce bias. The study had a few limitations. Diagnosis and treatment-related data (e.g., type of breast cancer, type and dosing of the chemotherapy agents, and phase of treatment) were not collected but may have influenced the study findings. Similarly, the timing of the interview (early versus late in the chemotherapy cycle) may have introduced a selection bias.

## Conclusion

The focus of this study was to explore the experience of women receiving chemotherapy and how they navigate physical activity participation during active treatment. The results of this study provide a richer understanding of the factors that influence physical activity participation among women affected by breast cancer and may explain why physical activity participation declines significantly during chemotherapy. It was evident from the study findings that there was a change in how physical activity was perceived, prioritized, and experienced during chemotherapy. Future research should explore how physical activity can be better defined and measured based on the unique needs and perspectives of women receiving chemotherapy for breast cancer.

## Supplementary Information

Below is the link to the electronic supplementary material.

ESM 1 DOCX (24.1 KB)

## Data Availability

No datasets were generated or analysed during the current study.
