# Supplementary material for: “It’s adjusting the mindset”: physical activity participation during chemotherapy
Source: Support Care Cancer. 2026 Apr 9;34(5):415. doi: 10.1007/s00520-026-10628-8 (PMC13061770; doi:10.1007/s00520-026-10628-8)
Supplement: Supplementary file 1 — DOCX (24.1 KB) [file 520_2026_10628_MOESM1_ESM.docx]

**Supplementary File 1**

**Project Title:** “It’s adjusting the mindset:" Physical activity participation during chemotherapy

**Reflexivity Activity** (completed prior to each interview)**:**

1. Reflexivity Prompts

- Recognize your own potential bias via reflection. Reflect on your thoughts, feelings, beliefs and opinions about physical activity during chemotherapy.
- Identify how your knowledge/beliefs may influence your beliefs about patient experience during chemotherapy. Your knowledge or involvement with patients in the past may also influence what you hear from participants and how you respond.

1. Interviewer Commitment to Reflexivity: “I recognize the lens through which I am viewing this interview and I will not let them influence my ability to hear or respond to the participants experience with physical activity participation during chemotherapy.”

Pre-Interview Checklist:

☐ Signed Participant Consent Form

☐ Completed Reflexivity Activity

☐ Recording device charged and sound check completed

**Introduction to Interview:**

Script: *“My name is _______. Thank you for taking the time to talk with me today. The purpose of this study is to understand* *how receiving chemotherapy for the treatment of breast cancer might influence physical activity participation. Do you have any questions about the study or the consent form you signed before we get started?”*

If the participant has any questions, answer them. Use language from the consent form to answer any consent related questions. Once all questions have been answered proceed to next script.

Script: “*There are a couple things I’d like you to remember: There is no right or wrong answer. This is a safe place where you can share your honest thoughts and feelings. The more honest you are with yourself the more I can learn from you. If you are uncomfortable with any question or you would prefer not to answer, please let me know and we can skip that question. I have a copy of your signed consent to participate in a recorded interview, however, can you please provide verbal confirmation that are you are willing to be audio recorded during this interview?”*

If the participant does not provide verbal consent to be audio-recorded, the interview cannot proceed. Thank the participant for their time and conclude the interview. If the participant provides verbal confirmation that the interview may be audio recorded proceed to the next script.

Script: *“Thank you for your willingness to participate and being recorded. I am going to start the recording now.”*

**START RECORDING NOW**

**Interview:**

Script: *“Today is [date] and I am speaking with [participant number]. I am going to be asking you questions about your experience of receiving chemotherapy for the treatment of breast cancer and how chemotherapy might influence your participation in physical activity.”*

| **Stem Questions** | **Follow-up questions** |
| --- | --- |
| “Please describe how your experience with chemotherapy has been so far…” | - “What do you find most challenging?” - “You said you felt ___, can you tell me more about that [feeling/ emotion]? - “I heard you say you ____, can you describe that in a little more detail?” |
| “Tell me about what activities you do during the day….” | - “How do you decide which tasks to prioritize?” - “How does the way you spend your time now differ from pre-chemotherapy?” “How is it the same?” - “On days you had ___, how did your day look different?” - “It sounds like you feel ___, what do you think contributes to that feeling? - “I heard you say ___, how do you feel that has impacted your ability to engage in ___?” |
| “In your own words, how would you define physical activity? | - “Based on the definition you have provided, how would you describe your current level of physical activity….” - “You said ___, can you share some specific ways that you ___” - “In what ways might your level of physical activity look different if you were not receiving chemotherapy?” - “On days you have ___, how does that change your level of physical activity?” - “When you feel ___, what do you think contributes to that feeling? |
| “When you consider your personal journey on chemotherapy, what would you change, if anything, with regard to physical activity participation? | - “What would motivate/ demotivate you to participate in physical activity during chemotherapy? - “What would you need to change in order for you to prioritize physical activity?” - “I heard you say ___, how do you feel that has impacted your ability to engage in ___?” |
| “Is there anything else you would like to share about your experience with physical activity during chemotherapy? | |

**NOTE:** The interview questions were drawn from the interview guide but remained flexible and adaptable based on the participant response to encourage participants to elaborate on their feelings, thoughts, and experiences.

**Post-Interview Reflection Prompts (completed at the conclusion of each interview):**

- What are your immediate thoughts, feelings, and impressions? What new or surprising concepts emerged from the interview?
- Consider your commitment to reflexivity. Were questions leading or experiences left unexplored?
